# Rapid Construction of a Chloromethyl-Substituted Duocarmycin-like Prodrug

**DOI:** 10.3390/molecules28124818

**Published:** 2023-06-16

**Authors:** Christoffer Bengtsson, Ylva Gravenfors

**Affiliations:** Drug Discovery & Development Platform, Science for Life Laboratory, Department of Organic Chemistry, Stockholm University, Tomtebodavägen 23a, 17165 Solna, Sweden; ylva.gravenfors@su.se

**Keywords:** duocarmycin, prodrug, selective halogenation, 1,2,3,6-tetrahydropyrrolo[3,2-*e*]indole

## Abstract

The construction of duocarmycin-like compounds is often associated with lengthy synthetic routes. Presented herein is the development of a short and convenient synthesis of a type of duocarmycin prodrug. The 1,2,3,6-tetrahydropyrrolo[3,2-*e*]indole-containing core is here constructed from commercially available Boc-5-bromoindole in four steps and 23% overall yield, utilizing a Buchwald–Hartwig amination followed by a sodium hydride-induced regioselective bromination. In addition, protocols for selective mono- and di-halogenations of positions 3 and 4 were also developed, which could be useful for further exploration of this scaffold.

## 1. Introduction

**Figure 1:**
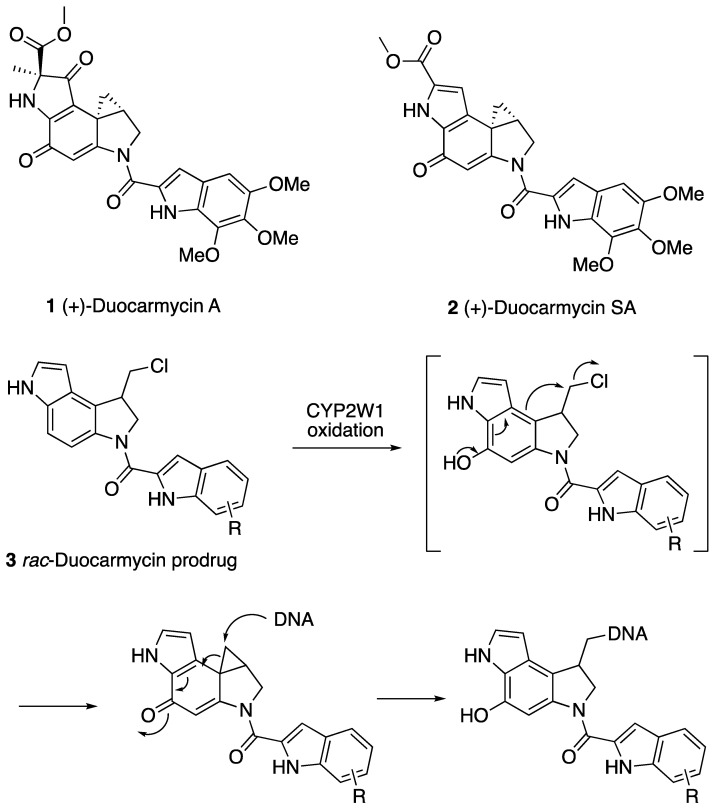
Structures of duocarmycin A, SA, and the duocarmycin prodrug with its activation by site-selective CYP2W1 oxidation.

**Figure 2:**
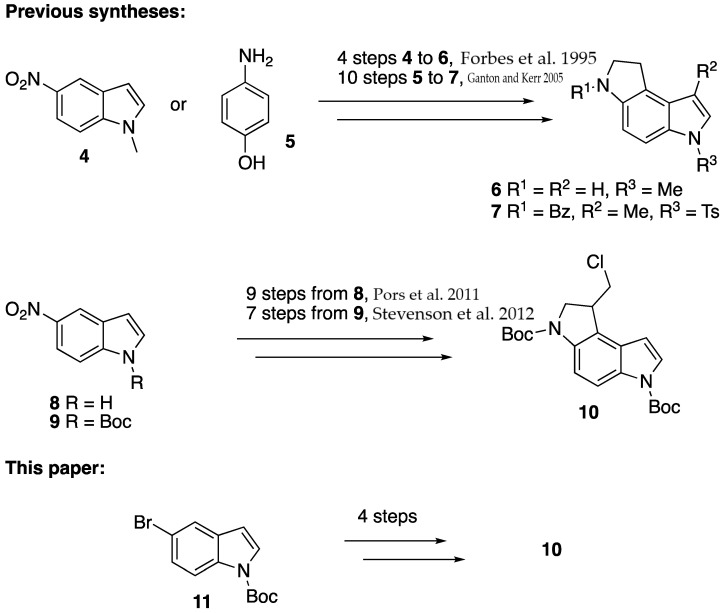
Previous versus new routes from commercial starting materials [14,16,21,22].

Duocarmycin A (**1**) and SA (**2**) are prominent members of the duocarmycin family that possess extreme cytotoxic properties (Figure 1) [1,2,3]. They were isolated from the *Streptomyces* sp. in Japan in 1988 and 1990, respectively [4,5]; in the early 1990s, their structures were confirmed by synthesis [6,7,8]. Since then, duocarmycin and its analogs have attracted a lot of attention among synthetic and medicinal chemists, owing to their structural complexity and interesting biological properties. Their mode of action is site-specific DNA alkylation, and their strongly alkylating properties can be attributed to the strained cyclopropane moiety (Figure 1). Unfortunately, the cytotoxicity is not only devoted to the cancer cells; therefore, a variety of duocarmycin analogs [1,2,3], prodrugs [9,10,11,12,13,14,15,16,17,18,19], and even antibody–drug conjugates [20] have been developed in the pursuit for more selective cancer treatments. In a medicinal chemistry project working with prodrugs that, upon site-selective CYP2W1 oxidation, form the phenolic counterpart and render the compound harmful [14,17] (**3**, Figure 1), we needed access to the chloromethyl-substituted 1,2,3,6-tetrahydropyrrolo[3,2-*e*]indole core **10** (Figure 2).

The existing synthetic pathways are elaborative and/or give the wrong substitution pattern (Figure 2). Furthermore, in our early attempts to use Boc-5-nitroindole **9** as starting material, we faced several problems, such as over-reduction when reducing the nitro group (i.e., the generation of indoline), the generation of complex mixtures when performing the halogenation reaction on the aniline, and problems with controlling the mono-Boc protection of the aniline.

**Figure 3:**
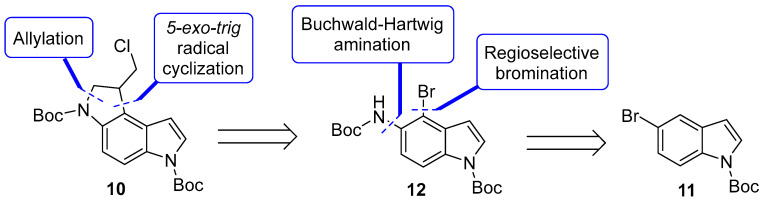
Retrosynthetic analysis.

In our approach, we envisioned that the desired di-Boc-protected 5-aminoindole intermediate **12** (Figure 3) could be synthesized from commercially available Boc-5-bromoindole **11** via a Buchwald–Hartwig amination with *t*Bu-carbamate followed by a regioselective bromination. This strategy would considerably shorten the route and also overcome the problems related to the nitro reduction and mono-Boc protection of the aniline nitrogen; vide supra.

## 2. Results and Discussion

**Scheme 1:**
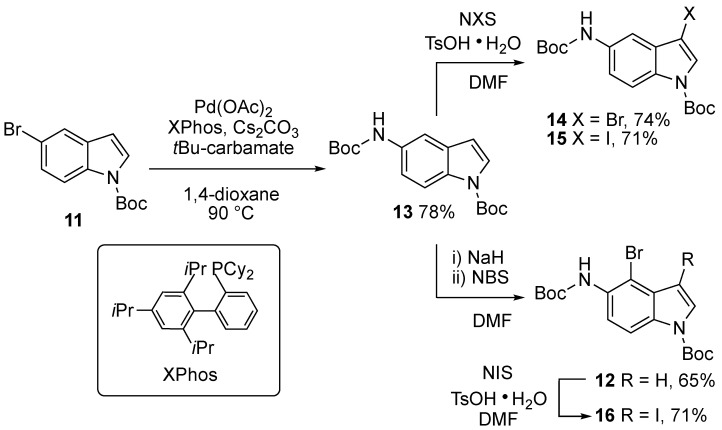
Buchwald–Hartwig amination and subsequent regioselective halogenations.

The Pd(OAc)_2_/XPhos-catalyzed Buchwald–Hartwig amination of Boc-5-bromoindole (**11**) with *t*Bu-carbamate performed well, and compound **13** could be isolated in 78% yield (Scheme 1). Performing the subsequent halogenation under acidic conditions (i.e., NXS/TsOH) on the Boc-protected aniline gave the wrong regioisomer, although with complete selectivity, and the 3-bromo (**14**) and 3-iodo (**15**) products could be isolated in 74% and 71% yields, respectively, using the two different halogen sources. We envisioned that the deprotonation of the Boc-protected aniline with NaH prior to the halogenation might render the aromatic ring sufficiently electron-rich to direct the halogenation to the right position (see Supporting Information). Gratifyingly, that strategy gave the desired 4-bromo analog **12** in 65% yield with complete regioselectivity. All attempts to introduce iodine in this position failed, even when using a more electrophilic I^+^ source (i.e., *N*-Iodosaccharin [23]), other solvents, or elevated temperatures.

**Scheme 2:**
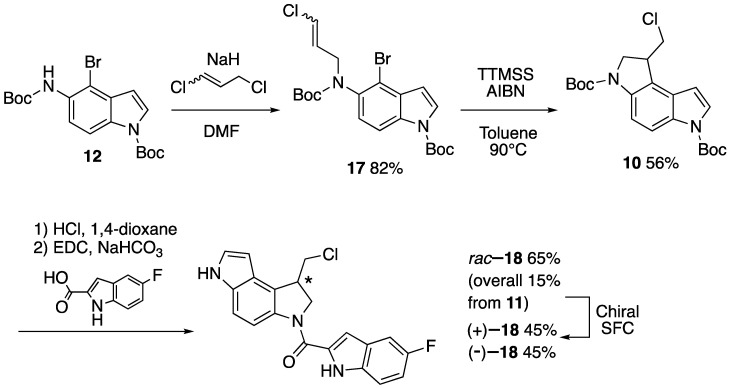
Synthesis of the duocarmycin-type prodrug, * denotes the chiral center.

To our delight, further halogenation of **12** to give 3-iodo-4-bromo compound **16** went smoothly under acidic conditions (NIS/TsOH) in 71% yield. To conclude the synthesis towards the duocarmycin-type prodrug, compound **12** smoothly underwent allylation with 1,3-dichloropropene to give **17 [14]** in 82% yield, followed by a tris(trimethylsilyl)silane (TTMSS)/azaisobutyronitrile (AIBN)-induced radical 5-*exo*-trig cyclization according to published procedures to furnish compound **10** [14] in 56% yield (Scheme 2). After Boc deprotection and subsequent EDC/NaHCO_3_ amide coupling with 5-fluoroindole-2-carboxylic acid, the desired prodrug *rac*—**18** [17] was isolated in 65% yield over two steps. In addition, the enantiomers were separated by chiral supercritical fluid chromatography (SFC) to give (+)—**18** and (−)—**18** with ee ≥ 99%.

## 3. Materials and Methods

General Methods: All solvents and reagents were used as received from commercial suppliers. *N*-Bromosuccinimide (NBS) was recrystallized from hot water and dried under vacuum for 24 h and then stored under cold and dark conditions. Sodium hydride was used as 60% dispersion in mineral oil. Column chromatography was employed on normal-phase silica gel (230–400 mesh, 60 Å; the eluents are given in brackets). ^1^H- and ^13^C-NMR spectra were recorded on a 400 MHz spectrometer at 298 K and calibrated using the residual peak of the solvent as an internal standard [CDCl_3_ (CHCl_3_ δ_H_ 7.26 ppm, CDCl_3_ δ_C_ 77.16 ppm)]. HRMS was performed using a microTOF instrument with electrospray ionization (ESI), and sodium formate was used as a calibration chemical. Optical rotations were measured on a polarimeter at 589 nm (D line of sodium) and 20 °C. Chiral chromatography was performed on supercritical fluid chromatography equipment, using mixtures of MeOH and supercritical CO_2_ as eluents.

Di-*tert*-butyl 1-(chloromethyl)-1,2-dihydropyrrolo[3,2-*e*]indole-3,6-dicarboxylate (**10**): *tert*-Butyl-4-bromo-5-((*tert*-butoxycarbonyl)(3-chloroallyl)amino)-1*H*-indole-1-carboxylate **17** (600 mg, 1.24 mmol) was dissolved in dry toluene (40 mL), and the solution was degassed for 1 h (by bubbling N_2_ gas through the solution under stirring). Azobisisobutyronitrile (AIBN) (49 mg, 0.30 mmol) and tris(trimethylsilyl)silane (TTMSS) (0.41 mL, 1.34 mmol) were added, and the reaction was heated to 90 °C (with a preheated oil bath) in a sealed tube for 5 h. The solvent was evaporated, and the crude material was dissolved in MeOH (12 mL) and stirred at rt for 10 min. The solvent was evaporated, and the crude product was purified by column chromatography on silica gel (hexanes:EtOAc 95:5) to give compound **10** as a colorless oil (280 mg, 56%). The spectral data agreed with the published data [14].

*tert*-Butyl 4-bromo-5-((*tert*-butoxycarbonyl)amino)-1*H*-indole-1-carboxylate (**12**): *tert*-Butyl 5-((*tert*-butoxycarbonyl)amino)-1*H*-indole-1-carboxylate **13** (200 mg, 0.60 mmol) was dissolved in dry DMF (2 mL) and cooled to 0 °C with an ice bath. NaH (60 mg, 60% in mineral oil, 1.5 mmol) was added, followed by NBS (129 mg, 0.72 mmol); the ice bath was removed, and the reaction was stirred for 30 min. The reaction mixture was poured onto saturated NaHCO_3_ (aq) and extracted with EtOAc. The organic phase was dried (Na_2_SO_4_), filtered, and concentrated. The crude material was purified by column chromatography on silica gel (hexanes:EtOAc 95:5) to give compound **12** as a colorless foam (160 mg, 65%). The spectral data agreed with the published data [14].

*tert*-Butyl 5-((tert-butoxycarbonyl)amino)-1*H*-indole-1-carboxylate (**13**): *N*-Boc-5-bromoindole **11** (1.5 g, 5.06 mmol), *tert*-butyl carbamate (712 mg, 6.08 mmol), Pd(OAc)_2_ (57 mg, 0.25 mmol), XPhos (241 mg, 0.50 mmol), and Cs_2_CO_3_ (2.31 g, 7.09 mmol) were mixed in dry 1,4-dioxane (45 mL), and the vessel was flushed with N_2_ gas, sealed, and heated to 90 °C for 20 h. The reaction mixture was diluted with EtOAc, filtered through Celite, and concentrated. The crude material was purified by column chromatography on silica gel (hexanes:EtOAc 95:5) to give compound **13** as a colorless foam (1.32 g, 78%). ^1^H-NMR (CDCl_3_, 400 MHz) δ 8.01 (brd, *J* = 8.0 Hz, 1H), 7.75 (brs, 1H), 7.55 (brd, *J* = 4.0 Hz, 1H), 7.14 (dd, *J* = 8.0, 4.0 Hz, 1H), 6.70 (brs, 1H, NH), 6.48 (dd, *J* = 3.7, 0.8 Hz, 1H), 1.65 (s, 9H), 1.52 (s, 9H); ^13^C-NMR (CDCl_3_, 100 MHz) δ 153.3, 149.8, 133.7, 131.5, 131.1, 126.6, 116.4, 115.3, 110.9, 107.4, 83.6, 80.3, 28.5 (3C), 28.3 (3C); HRMS (ESI/TOF) *m*/*z*: [M + Na]^+^ Calcd for C_18_H_24_N_2_O_4_Na 355.1634; Found 355.1633.

*tert*-Butyl 3-bromo-5-((tert-butoxycarbonyl)amino)-1*H*-indole-1-carboxylate (**14**): *tert*-Butyl 5-((tert-butoxycarbonyl)amino)-1*H*-indole-1-carboxylate **13** (200 mg, 0.60 mmol) was dissolved in DMF (2 mL), NBS (118 mg, 0.66 mmol) and TsOH·H_2_O (23 mg, 0.12 mmol) were added, and the reaction was stirred at rt for 10 min. The reaction mixture was poured onto saturated NaHCO_3_ (aq) and extracted with EtOAc. The organic phase was dried (Na_2_SO_4_), filtered, and concentrated. The crude material was purified by column chromatography on silica gel (hexanes:EtOAc 95:5) to give compound **14** as a colorless foam (183 mg, 74%). ^1^H-NMR (CDCl_3_, 400 MHz) δ 8.02 (brd, *J* = 8.0 Hz, 1H), 7.65 (brs, 1H), 7.60 (brs, 1H), 7.24 (brd, *J* = 8.0 Hz, 1H), 6.67 (brs, 1H, NH), 1.65 (s, 9H), 1.54 (s, 9H); ^13^C-NMR (CDCl_3_, 100 MHz) δ 153.1, 148.9, 134.5, 130.9, 130.0, 125.5, 117.5, 115.6, 109.2, 97.9, 84.4, 80.6, 28.5 (3C), 28.3 (3C); HRMS (ESI/TOF) *m*/*z*: [M + Na]^+^ Calcd for C_18_H_23_BrN_2_O_4_Na 433.0739; Found 433.0755.

*tert*-Butyl 5-((tert-butoxycarbonyl)amino)-3-iodo-1*H*-indole-1-carboxylate (**15**): *tert*-Butyl 5-((*tert*-butoxycarbonyl)amino)-1*H*-indole-1-carboxylate 13 (1.3 g, 3.91 mmol) was dissolved in DMF (14 mL), NIS (1.06 g, 4.71 mmol) and TsOH·H_2_O (149 mg, 0.78 mmol) were added, and the reaction was stirred at rt for 15 h. The reaction mixture was poured onto saturated NaHCO_3_ (aq) and extracted with EtOAc. The organic phase was washed with 10 wt% Na_2_S_2_O_5_ (aq), dried (Na_2_SO_4_), filtered, and concentrated. The crude material was purified by column chromatography on silica gel (hexanes:EtOAc 95:5) to give compound **15** as a colorless foam (1.28 g, 71%). ^1^H-NMR (CDCl_3_, 400 MHz) δ 8.00 (brd, *J* = 8.0 Hz, 1H), 7.69 (brs, 1H), 7.53–7.46 (m, 1H), 7.27 (brd, *J* = 8.0 Hz, 1H), 6.73 (brs, 1H, NH), 1.65 (s, 9H), 1.54 (s, 9H); ^13^C-NMR (CDCl_3_, 100 MHz) δ 153.1, 148.7, 134.6, 132.7, 131.1, 130.8, 117.5, 115.5, 111.3, 84.3, 80.6, 65.4, 28.5 (3C), 28.2 (3C); HRMS (ESI/TOF) *m*/*z*: [M + Na]^+^ Calcd for C_18_H_23_IN_2_O_4_Na 481.0601; Found 481.0595.

*tert*-Butyl 4-bromo-5-((*tert*-butoxycarbonyl)amino)-3-iodo-1*H*-indole-1-carboxylate (**16**): *tert*-Butyl 4-bromo-5-((*tert*-butoxycarbonyl)amino)-1*H*-indole-1-carboxylate **12** (140 mg, 0.34 mmol) was dissolved in DMF (1.4 mL), NIS (114 mg, 0.51 mmol) and TsOH·H_2_O (16 mg, 0.08 mmol) were added, and the reaction was stirred at rt for 16 h. The reaction mixture was poured onto saturated NaHCO_3_ (aq) and extracted with EtOAc. The organic phase was washed with 10 wt% Na_2_S_2_O_5_ (aq), dried (Na_2_SO_4_), filtered, and concentrated. The crude material was purified by column chromatography on silica gel (hexanes:EtOAc 95:5) to give compound **16** as a colorless foam (130 mg, 71%). ^1^H-NMR (CDCl_3_, 400 MHz) δ 8.11 (m, 2H), 7.77 (s, 1H), 7.08 (brs, 1H, NH), 1.65 (s, 9H), 1.54 (s, 9H); ^13^C-NMR (CDCl_3_, 100 MHz) δ 153.0, 148.2, 134.0, 132.7, 131.6, 126.5, 118.5, 114.5, 105.3, 85.0, 81.1, 61.2, 28.5 (3C), 28.2 (3C); HRMS (ESI/TOF) *m*/*z*: [M + Na]^+^ Calcd for C_18_H_22_BrIN_2_O_4_Na 558.9706; Found 558.9700.

*tert*-Butyl-4-bromo-5-((tert-butoxycarbonyl)(3-chloroallyl)amino)-1*H*-indole-1-carboxylate (**17**): *tert*-Butyl 4-bromo-5-((*tert*-butoxycarbonyl)amino)-1*H*-indole-1-carboxylate **12** (650 mg, 1.58 mmol) was dissolved in dry DMF (12 mL) and cooled to 0 °C, NaH (190 mg, 60% in mineral oil, 4.74 mmol) was added, and the reaction was stirred at 0 °C for 5 min. 1,3-Dichloropropene was added, the ice bath was removed, and the reaction was stirred at rt for 1 h. The reaction mixture was poured onto saturated NaHCO_3_ (aq) and extracted with EtOAc. The organic phase was dried (Na_2_SO_4_), filtered, and concentrated. The crude material was purified by column chromatography on silica gel (hexanes:EtOAc 95:5) to give compound **17** as a colorless oil (630 mg, 82%). The spectral data agreed with the published data [14].

(1-(chloromethyl)-1,6-dihydropyrrolo[3,2-*e*]indol-3(2H)-yl)(5-fluoro-1*H*-indol-2-yl)methanone (**18**): Di-*tert*-butyl 1-(chloromethyl)-1,2-dihydropyrrolo[3,2-*e*]indole-3,6-dicarboxylate **10** (280 mg, 0.69 mmol) was dissolved in 4 M HCl in 1,4-dioxane (15 mL, 60 mmol), and the reaction was stirred at rt for 22 h. The solvent was evaporated, and the crude material was coevaporated from EtOAc two times. The crude material, together with 5-fluoro-1*H*-indole-2-carboxylic acid **19** (148 mg, 0.83 mmol), *N*-ethyl-*N*′-(3-dimethylaminopropyl)carbodiimide hydrochloride (EDC) (396 mg, 2.07 mmol), and NaHCO_3_ (289 mg, 3.45 mmol), were mixed in dry DMF (10 mL), and the reaction was stirred at rt for 5 h. The reaction mixture was poured onto saturated NaHCO_3_ (aq) and extracted with EtOAc. The organic phase was dried (Na_2_SO_4_), filtered, and concentrated. The crude material was purified by column chromatography on silica gel (hexanes:EtOAc 60:40 to 50:50) to give compound **18** (253 mg, 65%) as an off-white solid. The spectral data agreed with the published results [17]. The racemic product was separated by chiral supercritical fluid chromatography (SFC) to give (+)—**18**, [α]_D_ (c = 1.0, acetone) +17 and (−)—**18**, [α]_D_ (c = 1.0, acetone) -17, both with ee ≥ 99% (for chromatographic conditions and chromatograms, see Supporting Information).

## 4. Conclusions

In conclusion, we developed a four-step route to the desired chloromethyl-substituted 1,2,3,6-tetrahydropyrrolo[3,2-*e*]indole core **10**, utilizing an unconventional NaH promoted site-selective bromination of Boc-protected amino indole **13** as the key step. Additionally, 3-iodo-4-bromo indole **16** constitutes an interesting starting point for further diversification. Closely related 3-iodo-4-bromo-indoles have been used in Pd-catalyzed cross-couplings such as the Mizoroki-Heck [24,25,26], Negishi [27], and Suzuki-Miyaura [28,29] reactions in various natural products and heterocyclic syntheses. Finally, the racemate of compound **18** was separated with chiral supercritical fluid chromatography for further investigation of this interesting prodrug.

## Data Availability

Not applicable.

## Supplementary Materials

Supporting information with ^1^H-NMR and ^13^C-NMR of all new compounds can be downloaded at: https://www.mdpi.com/article/10.3390/molecules28124818/s1.

## References

[1] Boger D.L., Johnson D.S. (1996). CC-1065 and the Duocarmycins: Understanding their Biological Function through Mechanistic Studies. Angew. Chem. Int. Ed. Engl..

[2] Boger D.L., Boyce C.W., Garbaccio R.M., Goldberg J.A. (1997). CC-1065 and the Duocarmycins: Synthetic Studies. Chem. Rev..

[3] Tercel M., Gieseg M.A., Denny W.A., Wilson W.R. (1999). Synthesis and Cytotoxicity of Amino-*seco*-DSA: An Amino Analogue of the DNA Alkylating Agent Duocarmycin SA. J. Org. Chem..

[4] Takahashi I., Takahashi K.-I., Ichimura M., Morimoto M., Asano K., Kawamoto I., Tomita F., Nakano H. (1988). Duocarmycin A, a new antitumor antibiotic from Streptomyces. J. Antibiot..

[5] Ichimura M., Ogawa T., Takahashi K.-I., Kobayashi E., Kawamoto I., Yasuzawa T., Takahashi I., Nakano H. (1990). Duocarmycin SA, a new antitumor antibiotic from *Streptomyces* sp.. J. Antibiot..

[6] Fukuda Y., Nakatani K., Ito Y., Terashima S. (1990). First total synthesis of *dl*-duocarmycin A. Tetrahedron Lett..

[7] Boger D.L., McKie J.A., Nishi T., Ogiku T. (1996). Enantioselective Total Synthesis of (+)-Duocarmycin A, epi-(+)-Duocarmycin A, and Their Unnatural Enantiomers. J. Am. Chem. Soc..

[8] Boger D.L., Machiya K. (1992). Total synthesis of (+)-duocarmycin SA. J. Am. Chem. Soc..

[9] Tietze L.F., Schuster H.J., Schmuck K., Schuberth I., Alves F. (2008). Duocarmycin-based prodrugs for cancer prodrug monotherapy. Bioorg. Med. Chem..

[10] Li L.-S., Sinha S.C. (2009). Studies toward the duocarmycin prodrugs for the antibody prodrug therapy approach. Tetrahedron Lett..

[11] Schuster H.J., Krewer B., Von Hof J.M., Schmuck K., Schuberth I., Alves F., Tietze L.F. (2010). Synthesis of the first spacer containing prodrug of a duocarmycin analogue and determination of its biological activity. Org. Biomol. Chem..

[12] Lajiness J.P., Robertson W.M., Dunwiddie I., Broward M.A., Vielhauer G.A., Weir S.J., Boger D.L. (2010). Design, Synthesis, and Evaluation of Duocarmycin O-Amino Phenol Prodrugs Subject to Tunable Reductive Activation. J. Med. Chem..

[13] Tietze L.E., Schmuck K., Schuster H.J., Müller M., Schuberth I. (2011). Synthesis and Biological Evaluation of Prodrugs Based on the Natural Antibiotic Duocarmycin for Use in ADEPT and PMT. Chem. Eur. J..

[14] Pors K., Loadman P.M., Shnyder S.D., Sutherland M., Sheldrake H.M., Guino M., Kiakos K., Hartley J.A., Searcey M., Patterson L.H. (2011). Modification of the duocarmycin pharmacophore enables CYP1A1 targeting for biological activity. Chem. Commun..

[15] Wolfe A.L., Duncan K.K., Parelkar N.K., Weir S.J., Vielhauer G.A., Boger D.L. (2012). A Novel, Unusually Efficacious Duocarmycin Carbamate Prodrug That Releases No Residual Byproduct. J. Med. Chem..

[16] Stevenson R.J., Denny W.A., Tercel M., Pruijn F.B., Ashoorzadeh A. (2012). Nitro *seco* Analogues of the Duocarmycins Containing Sulfonate Leaving Groups as Hypoxia-Activated Prodrugs for Cancer Therapy. J. Med. Chem..

[17] Sheldrake H.M., Travica S., Johansson I., Loadman P.M., Sutherland M., Elsalem L., Illingworth N., Cresswell A.J., Reuillon T., Shnyder S.D. (2013). Re-engineering of the Duocarmycin Structural Architecture Enables Bioprecursor Development Targeting CYP1A1 and CYP2W1 for Biological Activity. J. Med. Chem..

[18] Uematsu M., Brody D.M., Boger D.L. (2015). A five-membered lactone prodrug of CBI-based analogs of the duocarmycins. Tetrahedron Lett..

[19] Giddens A.C., Lee H.H., Lu G.-L., Miller C.K., Guo J., Phillips G.D.L., Pillow T.H., Tercel M. (2016). Analogues of DNA minor groove cross-linking agents incorporating aminoCBI, an amino derivative of the duocarmycins: Synthesis, cytotoxicity, and potential as payloads for antibody–drug conjugates. Bioorg. Med. Chem..

[20] Menderes G., Bonazzoli E., Bellone S., Black J., Altweger G., Masserdotti A., Pettinella F., Zammataro L., Buza N., Hui P. (2017). SYD985, a novel duocarmycin-based HER2-targeting antibody-drug conjugate, shows promising antitumor activity in epithelial ovarian carcinoma with HER2/Neu expression. Gynecol. Oncol..

[21] Forbes I.T., Ham P., Booth D.H., Martin R.T., Thompson M., Baxter G.S., Blackburn T.P., Glen A., Kennett G.A., Wood M.D. (1995). 5-Methyl-1-(3-pyridylcarbamoyl)-1,2,3,5-tetrahydropyrrolo[2,3-*f*]indole: A Novel 5-HT2C/5-HT2B Receptor Antagonist with Improved Affinity, Selectivity, and Oral Activity. J. Med. Chem..

[22] Ganton M.D., Kerr M.A. (2005). A Domino Amidation Route to Indolines and Indoles: Rapid Syntheses of Anhydrolycorinone, Hippadine, Oxoassoanine, and Pratosine. Org. Lett..

[23] Dolenc D. (2000). N-Iodosaccharin—A New Reagent for Iodination of Alkenes and Activated Aromatics. Synlett.

[24] Harrington P.J., Hegedus L.S. (1984). Palladium-catalyzed reactions in the synthesis of 3- and 4-substituted indoles. Approaches to ergot alkaloids. J. Org. Chem..

[25] Harrington P.J., Hegedus L.S., McDaniel K.F. (1987). Palladium-catalyzed reactions in the synthesis of 3- and 4-substituted indoles. 2. Total synthesis of the N-acetyl methyl ester of (+/−)-clavicipitic acids. J. Am. Chem. Soc..

[26] Hegedus L.S., Toro J.L., Miles W.H., Harrington P.J. (1987). Palladium-catalyzed reactions in the synthesis of 3- and 4-substituted indoles. 3. Total synthesis of (+/−)-aurantioclavine. J. Org. Chem..

[27] Hegedus L.S., Sestrick M.R., Michaelson E.T., Harrington P.J. (1989). Palladium-catalyzed reactions in the synthesis of 3- and 4-substituted indoles. 4. J. Org. Chem..

[28] Hellal M., Singh S., Cuny G.D. (2012). Synthesis of Tetracyclic Indoles via Intramolecular α-Arylation of Ketones. J. Org. Chem..

[29] Chen K.X., Vibulbhan B., Yang W., Sannigrahi M., Velazquez F., Chan T.-Y., Venkatraman S., Anilkumar G.N., Zeng Q., Bennet F. (2012). Structure–Activity Relationship (SAR) Development and Discovery of Potent Indole-Based Inhibitors of the Hepatitis C Virus (HCV) NS5B Polymerase. J. Med. Chem..

