# Supplementary material for: Rapid Construction of a Chloromethyl-Substituted Duocarmycin-like Prodrug
Source: Molecules. 2023 Jun 16;28(12):4818. doi: 10.3390/molecules28124818 (PMC10304315; doi:10.3390/molecules28124818)
Supplement: Supplementary file 1 [file molecules-28-04818-s001.zip › molecules-2443998-supplementary.pdf]

Rapid Construction of a Chloromethyl-Substituted Duocarmycin-like Prodrug

Christoffer Bengtsson and Ylva Gravenfors

Drug Discovery and Development Platform, Science for Life Laboratory, Department of Organic Chemistry, Stockholm University, Tomtebodavägen 23a, 17165 Solna, Sweden

^{1}H-NMR shift prediction of compound **13**……………………………………………….....S2

^{1}H- and ^{13}C-NMR spectra of new compounds………………………..…….……..S3-S6

Chiral SFC chromatograms of (±)−**18**, (+)−**18** and (-)−**18**…..…………………………..S7

(1) 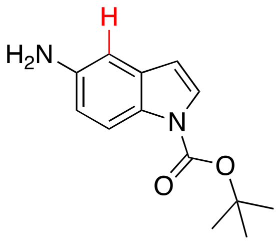 Predicted ^{1}H-NMR shift δ 7.53 ppm

(2) 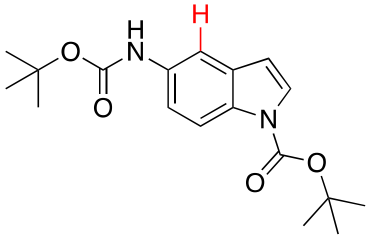 Predicted ^{1}H-NMR shift δ 8.48 ppm

(3) 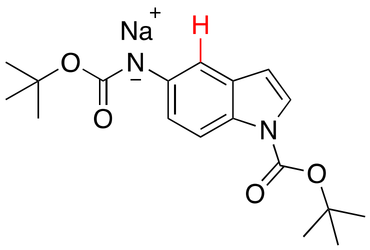 Predicted ^{1}H-NMR shift δ 7.63 ppm

^{1}H-NMR chemical shift predictions reveal that the proton in position 4 (highlighted in red) in the deprotonated species (3) experience similar electron density as the corresponding aniline (1). Predictions made in ChemDraw Professional version 16.0.0.82 with $CDCl_3$ as solvent.

^{1}H- and ^{13}C-NMR of compound **13** in $CDCl_3$

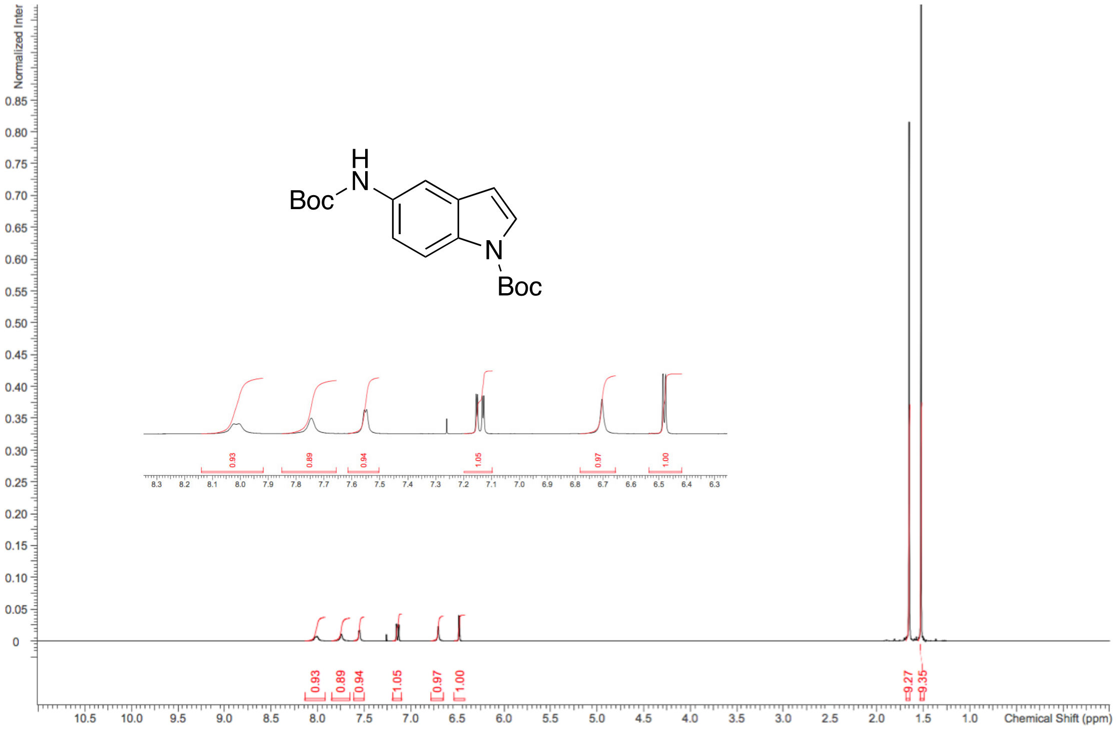

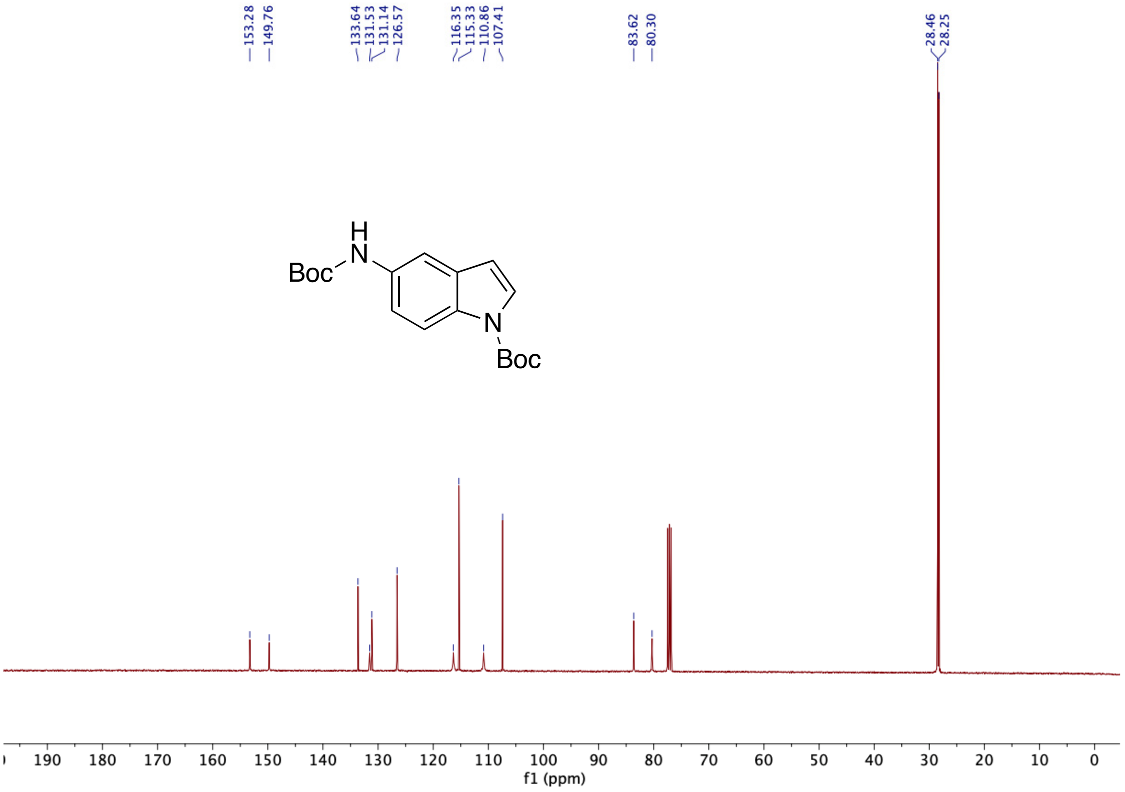

^{1}H- and ^{13}C-NMR of compound **14** in $CDCl_3$

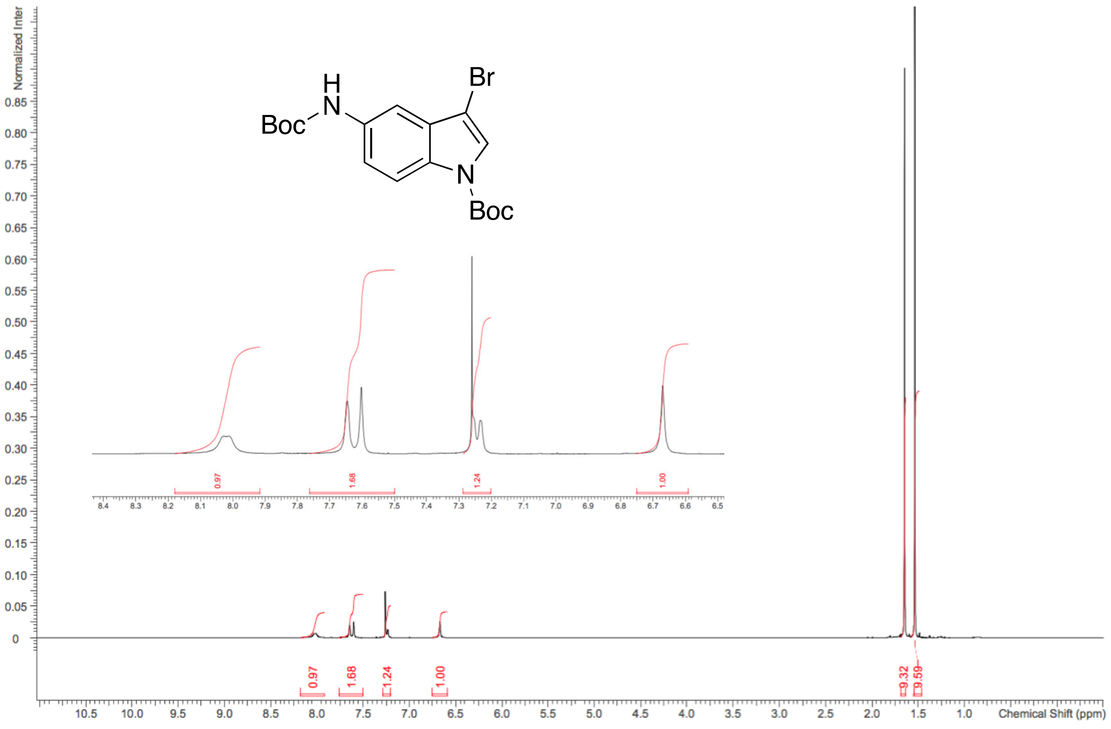

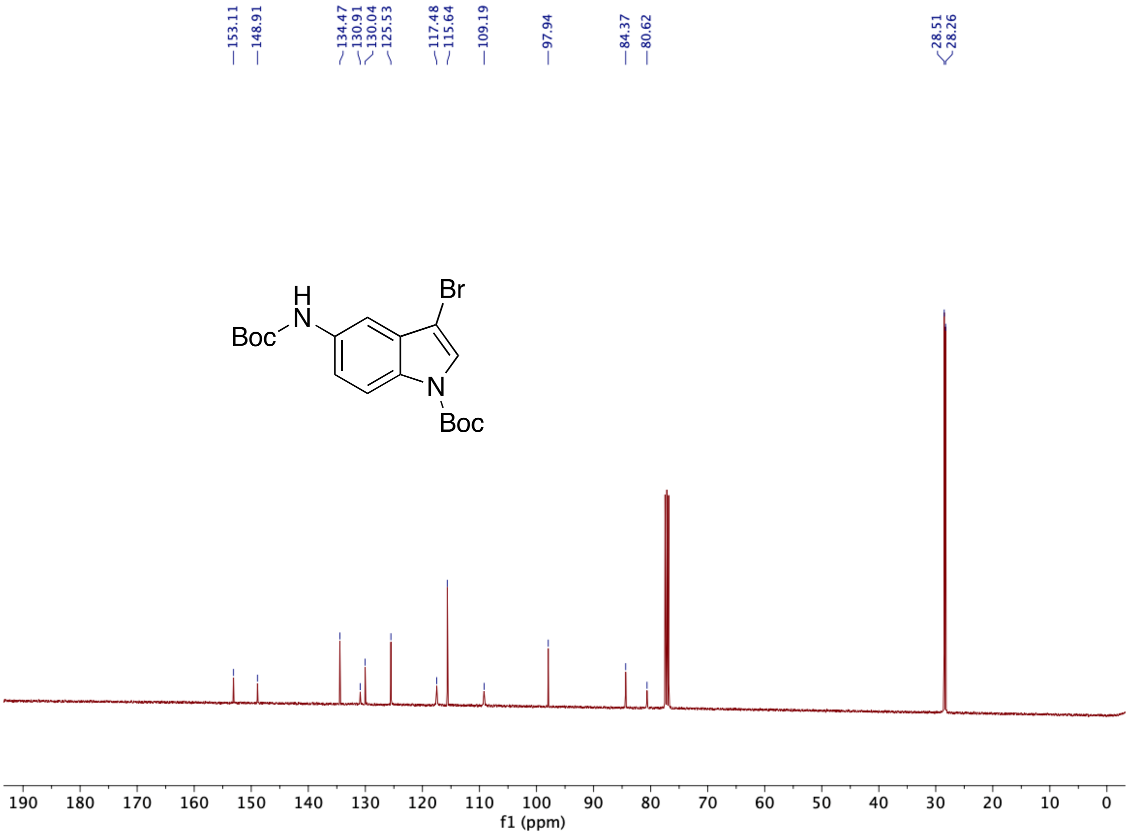

^{1}H- and ^{13}C-NMR of compound **15** in $CDCl_3$

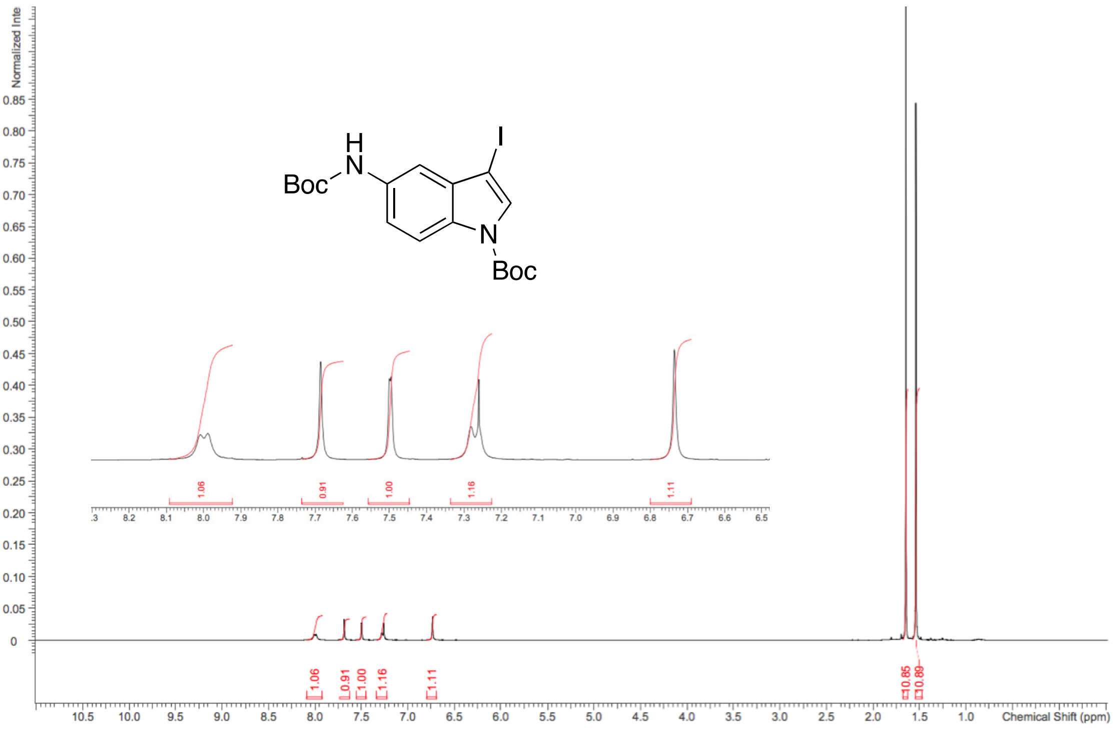

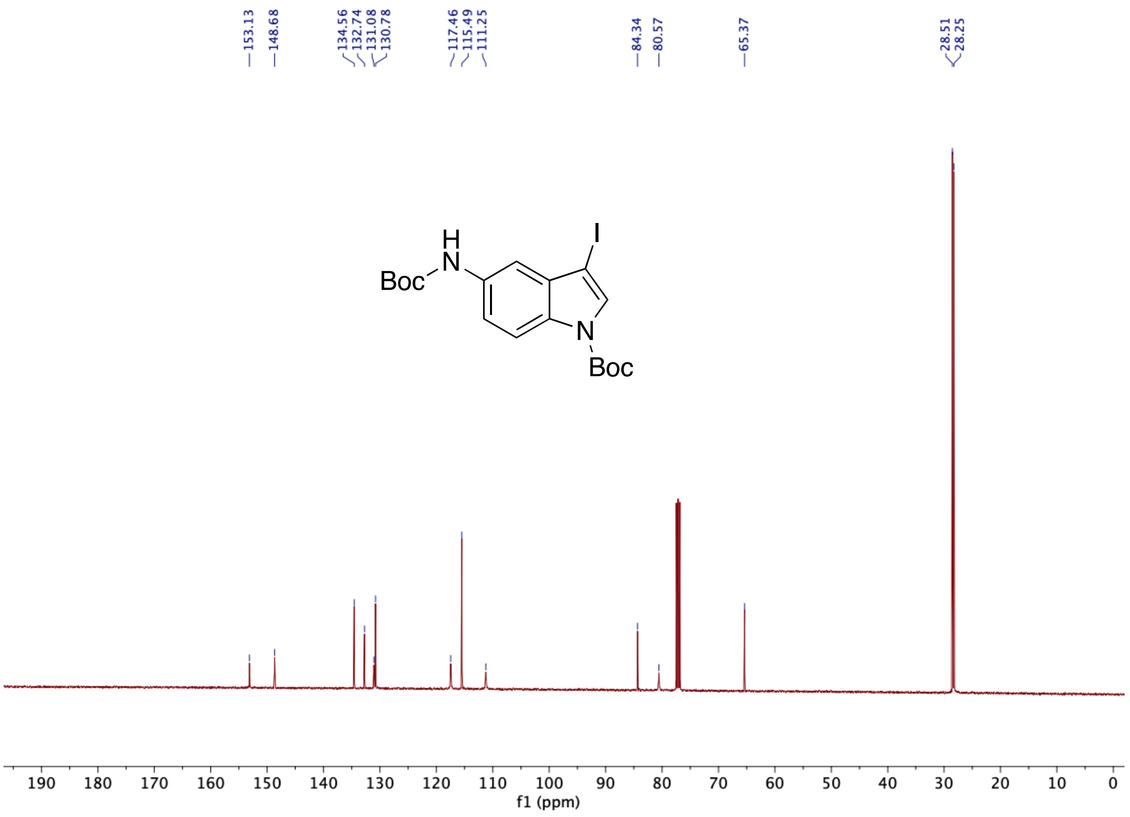

^{1}H- and ^{13}C-NMR of compound **16** in $CDCl_3$

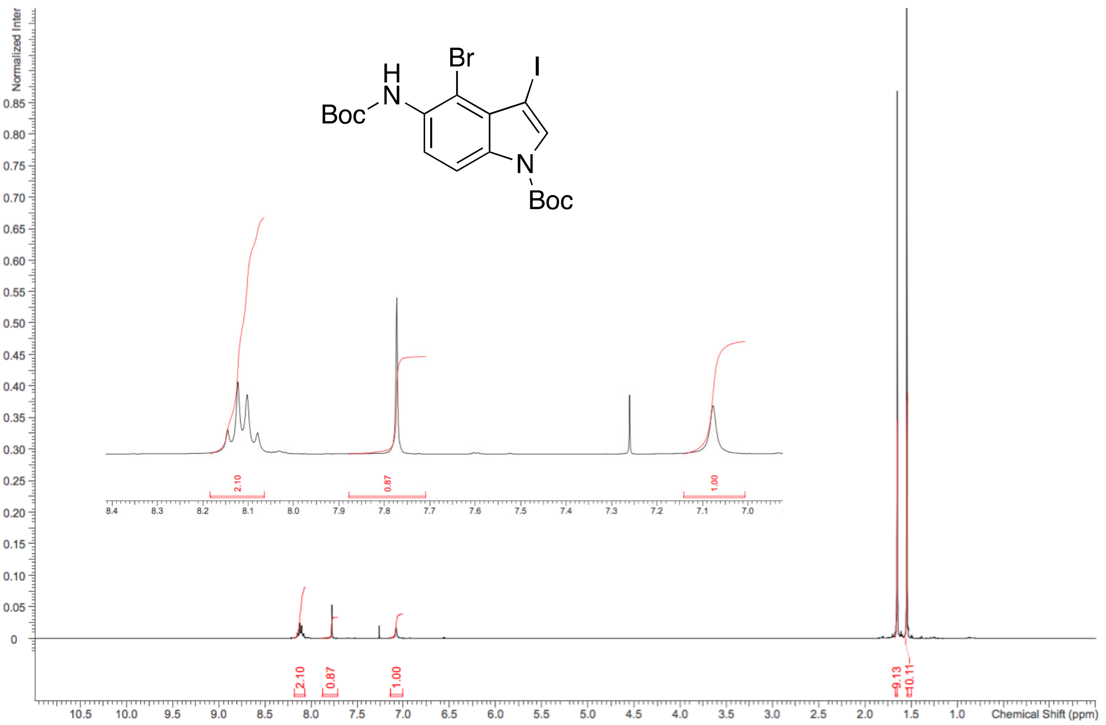

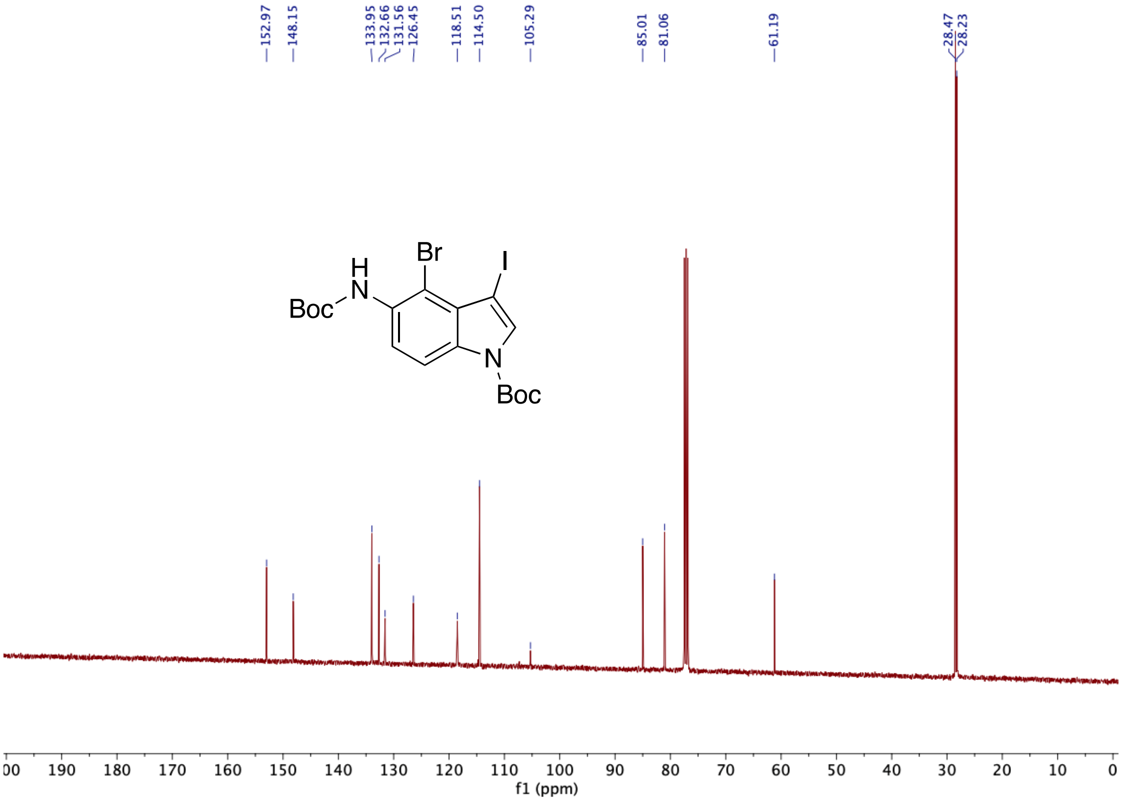

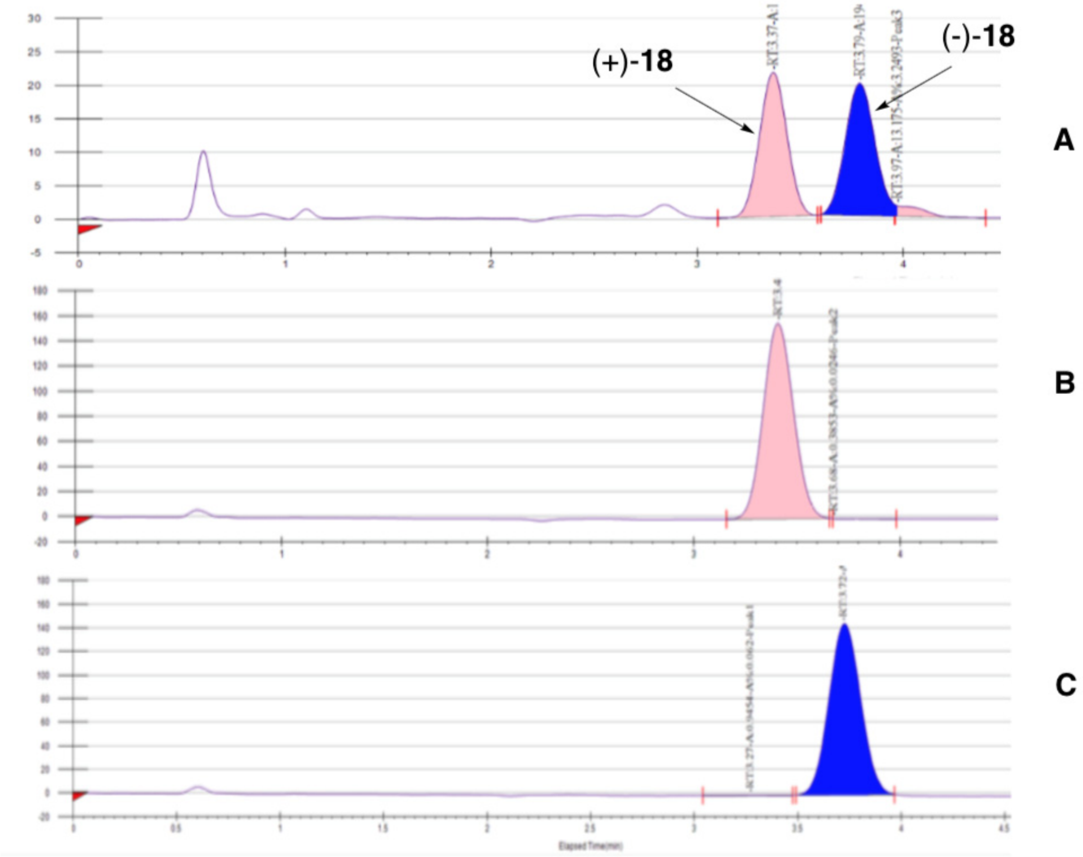

Chiral SFC separation of *rac*-**18**, Column: Chiralart Cellulose SB (10x250 mm, 5µm), Eluent: isocratic 40% MeOH in supercritical CO_2, Flowrate: 10 ml/min.
A: (±)-**18**; **B**: (+)-**18** [α]$_{589}$ +17 (c = 1.0, acetone), ee ≥ 99%; **C**: (-)-**18** [α]$_{589}$ -17 (c = 1.0, acetone), ee ≥ 99%.
